# Update on cardiomyopathies and sudden cardiac death

**DOI:** 10.1080/20961790.2019.1631957

**Published:** 2019-08-19

**Authors:** Stefania Rizzo, Elisa Carturan, Monica De Gaspari, Kalliopi Pilichou, Gaetano Thiene, Cristina Basso

**Affiliations:** Cardiovascular Pathology, Department of Cardio-Thoracic-Vascular Sciences & Public Health and Azienda Ospedaliera, University of Padua Medical School, Padua, Italy

**Keywords:** Forensic sciences, forensic pathology, autopsy, cardiomyopathies, genetics, sudden death

## Abstract

Sudden cardiac death (SCD) remains a leading mode of death in western countries. Since SCD can be the first and last clinical presentation of the underlying disease, autopsy could be the only medical examination available for early diagnosis and it should be performed according to the guidelines of the Association for European Cardiovascular Pathology. Although the vast majority of SCD are due to coronary artery disease, non-ischemic causes of SCD do exist and are prevalent in young people with structural (i.e. arrhythmogenic, hypertrophic and inflammatory cardiomyopathy) and non-structural (ion channel diseases) cardiomyopathies, accounting for up to one half of cases. A standardized autopsy protocol, in combination with blood sampling to ensure feasibility of postmortem molecular testing if needed, is mandatory. The pathologist is called to provide the correct diagnosis and to advice the relatives on the need of a cascade clinical and genetic screening in the presence of a heredo-familial disease.

## Introduction

**Figure 1.**
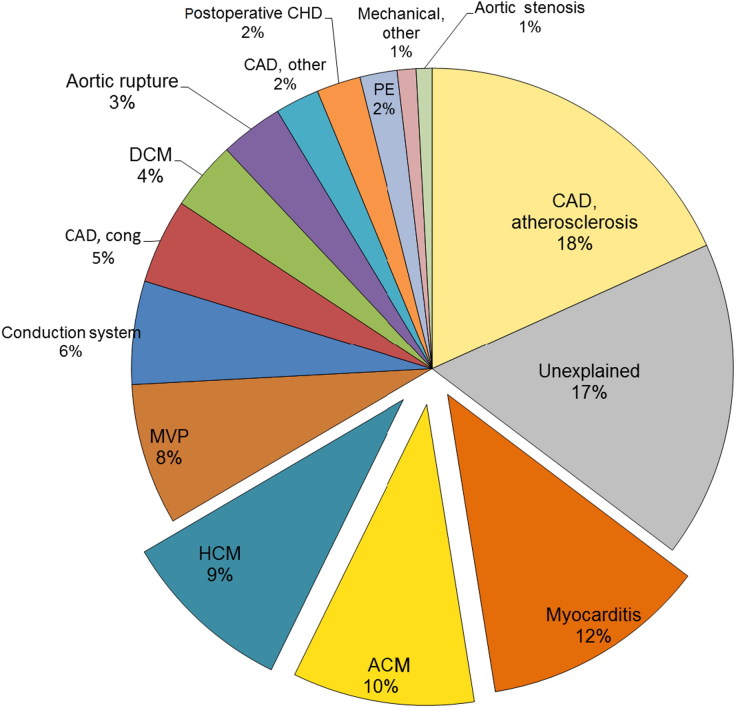
Causes of sudden death in Veneto Region Northeast Italy, time interval 1980–2013. CAD: coronary artery disease; ACM: arrhythmogenic cardiomyopathy; HCM: hypertrophic cardiomyopathy; MVP: mitral valve prolapse; cong: congenital anomalies of the coronary arteries; DCM: dilated cardiomyopathy; CHD: congenital heart disease; PE: pulmonary embolism.

Sudden death (SD) is mostly due to cardiovascular disease. Causes may be categorized into structural (e.g. great arteries, coronary arteries, myocardial, valvular and conduction system diseases) and non-structural diseases (channelopathies) [1] (Figure 1). The incidence of SD in the general population increases with age (about 0.01‰ per year in people <35 years, 1‰ per year in the age range 35–40 years, 2‰ per year by 60 years and 25% per year in the elderly [1–5]. According to the data collected in the Veneto Region, Northeast Italy, incidence of SD was significantly higher in young athletes compared to non-athletic young people (0.23% year *vs.* 0.09% per year; 2.5 fold more frequent), indicating that sport activity can be a risk factor in people affected by concealed cardiac disorders [6].

When performing autopsy investigation according to the European guidelines of cardiac SD [2], atherosclerotic coronary artery disease remains the main cause in older people, while about half of SD cases in the young population can be attributed to cardiomyopathies [1,7–9] (Figure 1).

The definition of cardiomyopathy evolved through times, along with the discovery of the pathogenic mechanisms, including the molecular basis, and the progresses linked to the new diagnostic techniques. After the original classification of cardiomyopathies published in the 1980s [10,11], the identification of novel clinico-pathological entities and of the genetic basis of these diseases raised the need of a genomic classification, distinguishing cytoskeleton (e.g., dilated cardiomyopathy), desmosomal (arrhythmogenic (ACM)), sarcomeric (as hypertrophic (HCM) and restrictive) and ion channel (channelopathies, e.g., long QT (LQT) or short QT (SQT) syndrome, Brugada syndrome and catecholaminergic polymorphic ventricular tachycardia (CPVT)) cardiomyopathies [12].

In 2006, the American Heart Association (AHA) proposed a new definition and classification [13]: “cardiomyopathies are a heterogenous group of diseases of the myocardium associated with mechanical and/or electrical dysfunction, which usually (but not invariably) exhibit inappropriate ventricular hypertrophy or dilatation and are due to a variety of causes that frequently are genetic” [13]. If myocardial dysfunction was caused by other cardiovascular abnormalities, such as valvular heart disease, systemic hypertension, congenital heart disease, and atherosclerotic coronary artery disease, these were excluded from the group of cardiomyopathies. Furthermore, the AHA distinguished primary and secondary cardiomyopathies based on the disorder being predominantly confined to heart muscle or part of a generalized systemic disease [13]. Primary cardiomyopathies were then subdivided into: (i) genetic cardiomyopathies (HCM, ACM, non-compaction, glycogen storage, Lenègre disease, mitochondrial, the channelopathies such as LQT and SQT syndromes, Brugada syndrome, CPVT); (ii) mixed cardiomyopathies (dilated or restrictive); and (iii) acquired cardiomyopathies (inflammatory, peripartum, tachycardia-induced, Tako-Tsubo).

In 2008, the European Society of Cardiology Working Group on Myocardial and Pericardial Diseases published a new position statement as an update on the classification. Cardiomyopathies were defined as “myocardial disorders in which the heart muscle is structurally and functionally abnormal, and in which coronary artery disease, hypertension, valvular and congenital heart disease are absent or do not sufficiently explain the observed myocardial abnormality”. Five types of cardiomyopathies are recognized according to the morphofunctional phenotype (HCM, dilated, ACM, restrictive and unclassified), each phenotype is then sub-classified into familial/genetic and non-familial/non-genetic forms [14].

More recently, in 2013, the World Heart Federation supported a new nosology system termed “MOGE(S)”, which aims to describe cardiomyopathies integrating phenotype description, information about extracardiac involvement, transmission pattern and genotype. Taking inspiration from TNM staging system for tumors, this nosological system describes a cardiomyopathy with five attributes: M: morphofunctional phenotype; O: involved organs; G: genetic/familial disease (or not familial); E: aetiology (genetic or not); S: functional status. The “S” component is particularly useful when the subjects are healthy carriers of the mutation or if they show initial modifications at imaging, suggesting the cardiomyopathy [15].

When dealing with cardiomyopathies at risk of SD, both structural (HCM and ACM) and non-structural (mostly channelopathies) diseases are considered. SD can occur also in other cardiomyopathies (dilated and restrictive cardiomyopathy), but they unlikely cope with effort performance because of failure symptoms, and are an exceptional cause of SD in the young and athletes.

### HCM

HCM is the most common genetically determined primary heart muscle disease, affecting 0.2%–0.5% of the general population [16]. It is characterized by extreme heterogeneity with regard to phenotypic expression, pathophysiology and clinical course. The pattern of inheritance is autosomal dominant, with the genetic basis residing in mutations of genes encoding sarcomeric proteins. A pathogenic mutation can be identified in 35%–45% of the probands affected by the disease and up to 65% when the family history is positive [17,18]. Of patients with positive genotype, about 70% have mutations in two genes, β-myosin heavy chain (*MYH7*) and myosin-binding protein C (*MYBPC3*). Troponin T (*TNNT2*) and several other genes each account for 5% or less of cases [19–21]. Due to genetic heterogeneity and variable phenotypes, the relation between sarcomere mutations and clinical outcome in patients with HCM has been proved unreliable.

In patients with HCM, absolute left ventricular wall thickness ranges widely from mild (13–15 mm) to massive (>50 mm). Morphologically, HCM may show either a symmetrical or, more frequently, asymmetrical pattern of left ventricular hypertrophy. In rare cases, HCM may present with unusual patterns of hypertrophy (e.g. apical hypertrophy) [22–25].

**Figure 2.**
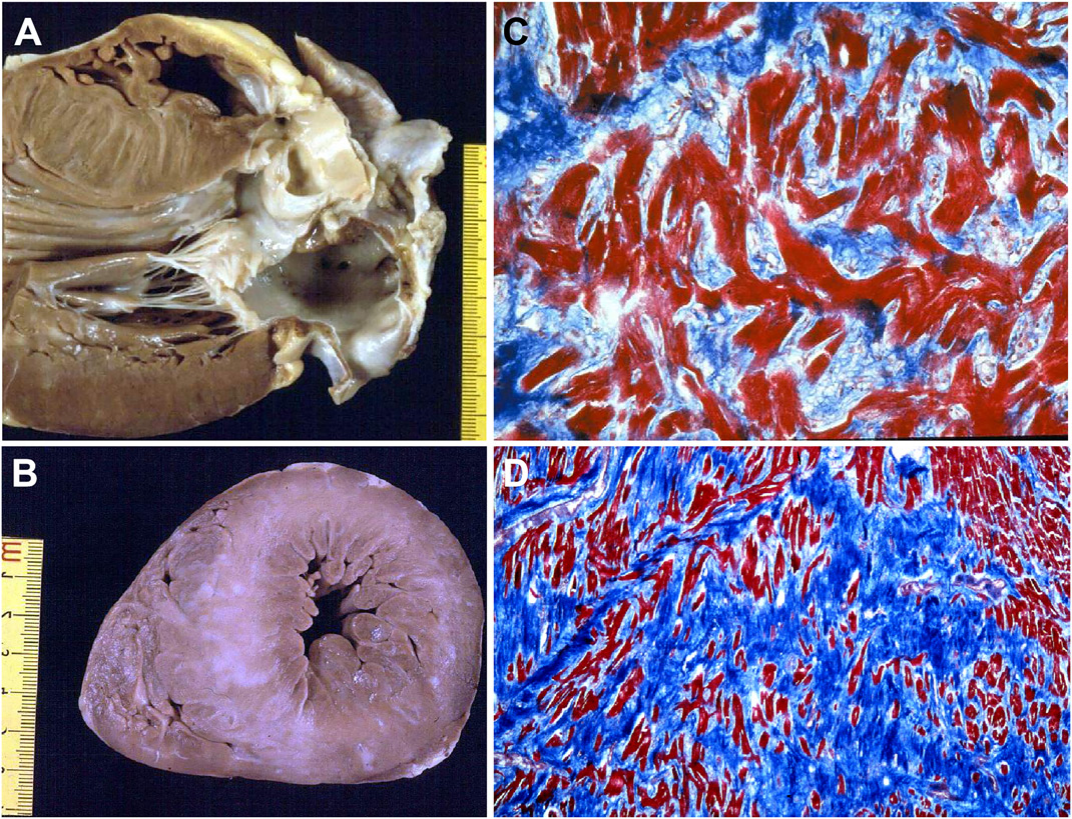
Arrhythmic sudden death in a 23-year-old man due to hypertrophic cardiomyopathy [24]. (A) Long axis view of the left ventricular outflow tract: the bulging of septal hypertrophy creates subaortic stenosis, which is aggravated by a fibrous plaque superimposed to the septal endocardium facing the anterior leaflet of the mitral valve; (B) Mid-apical cross-section of the same heart: note the asymmetric septal hypertrophy with reduced left ventricular cavity and the presence of white scars in the septum; (C) Fascicular disarray of the myocardium (Trichrome Heidenhain, ×120); (D) Disarray of single myocytes (Trichrome Heidenhain, ×30).

In the asymmetrical septal variant of HCM, thickening of the basal anterior septum with subaortic bulging leading to left ventricular outflow tract obstruction (Figure 2), with or without septal endocardial plaques (“friction lesion”), is observed. Many patients with HCM achieve normal life expectancy with little or no disability. However, SD can occur in asymptomatic patients and the disease can occasionally progress to heart failure (end-stage HCM) resulting in a markedly dilated left ventricular cavity [26].

**Figure 3.**
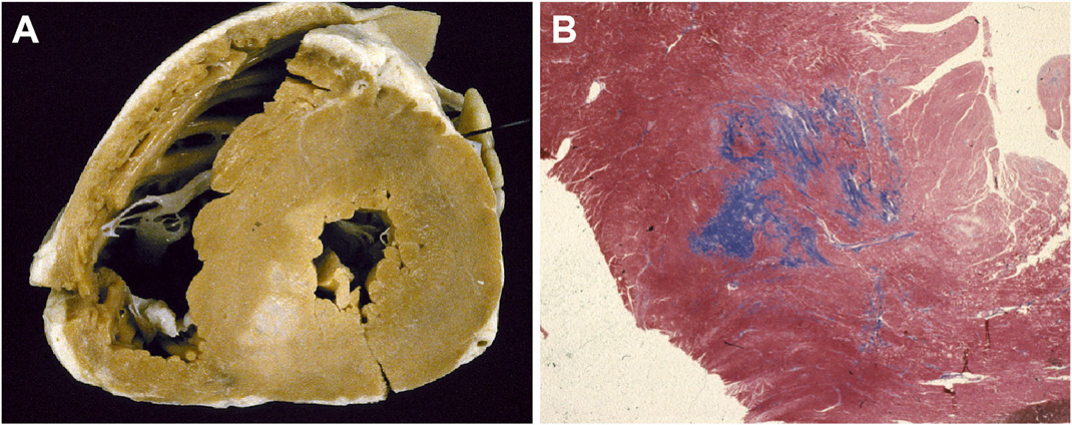
Arrhythmic sudden death on effort in a 14-year-old boy due to hypertrophic cardiomyopathy. (A) Cross-section of the heart shows a large focus of fibrosis in the postero-septal wall of left ventricle. Note the intramyocardial course of the left anterior descending coronary artery; (B) Histological examination of the ventricular septum confirms the large area of replacement-type fibrous tissue, most probably the result of previous ischaemic injury (Trichrome Heidenhain, ×3).

Myocardial bridge, i.e. a deep intramyocardial course of the left anterior descending coronary artery, is much more frequent in HCM than in the normal heart (Figure 3) [27–29]. However, a significant relation between myocardial bridge and SD has not been definitely proved in HCM [27].

The final diagnosis of HCM implies exclusion of “secondary” forms of left ventricular hypertrophy, including Fabry disease, glycogen storage disease and mitochondrial cardiomyopathies [19]. Finally, HCM must be differentiated from the exercise-induced hypertrophy (so-called “athlete’s heart”), which can be found in more than one-third of highly trained athletes presenting as an enlarged left ventricular cavity with increased wall thickness up to 13–14 mm [1].

Myocyte hypertrophy, myocardial disarray, and interstitial fibrosis are histopathologic features of HCM [24–27] (Figures 2 and 3). Myocyte disarray consists of architectural disorganization of the cardiomyocytes, either single or in fascicles, with perpendicular or oblique alignment to each other [22]. However, myocyte disarray is not pathognomonic of HCM, and can be observed also in congenital heart diseases and in normal adult hearts, although usually mild and confined to the ventricular free wall-septal junctions. At higher magnification, the myocytes are hypertrophied with nuclear pleomorphism and hyperchromasia. Intramural small vessel disease is another typical histologic feature of HCM, with variable degree of lumen stenosis attributed to intimal smooth muscle cell hyperplasia and medial hypertrophy or fibrosis [24,25,27].

Ischaemic myocardial injury, in the form of either acute/subacute myocyte necrosis or chronic fibrous scars has been reported in young SD victims [24] (Figures 2 and 3).

**Figure 4.**
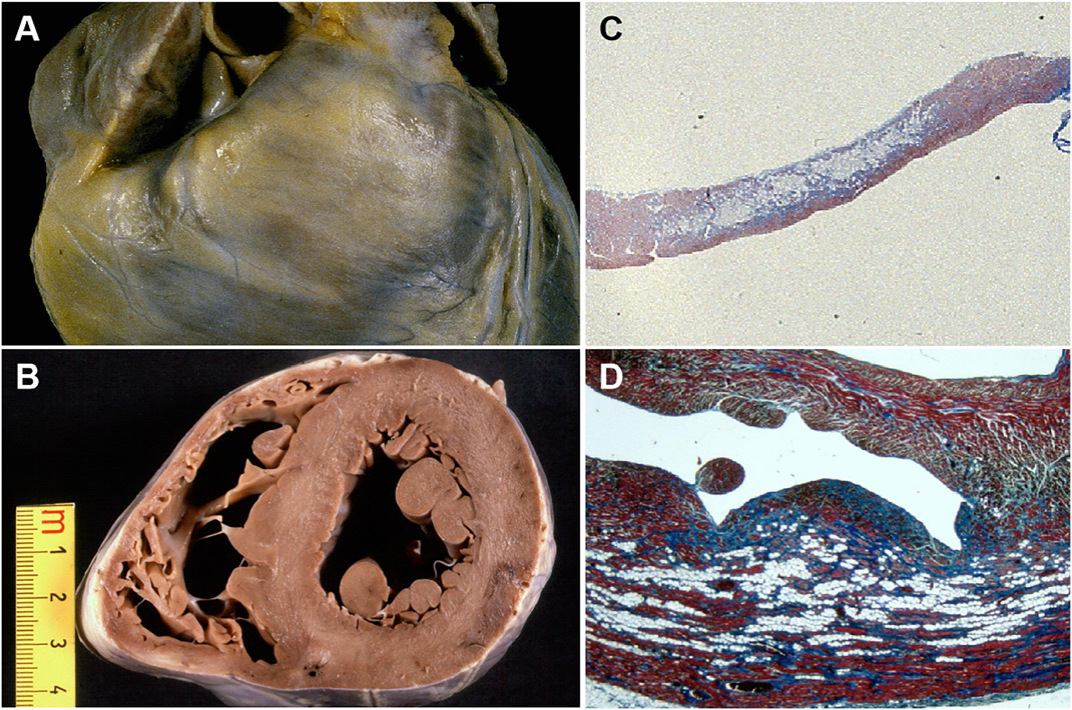
Arrhythmic sudden death due to arrhythmogenic cardiomyopathy (segmental form) in a 26-year-old athlete. (A) Anterior view of the right ventricular outflow tract which appears mildly dilated; (B) Cross-section of the heart showing the absence of right ventricular free wall aneurysms: note the spotty involvement of the posterior right ventricular free wall; (C) Histology of the right ventricular outflow tract: note the regional loss of myocardium with fibro-fatty replacement (Trichrome Heidenhain, ×2.5); (D) Histology of the posterior right ventricular free wall: note the fibro-fatty replacement of the myocardium in the absence of wall thinning (Trichrome Heidenhain, ×5).

Contrast enhanced cardiac magnetic resonance (CMR) can detect these scars *in vivo*, so that it has been proposed as an additional tool for risk stratification in HCM patients [29].

The malignant ACM substrate in HCM originates from the combination of myocardial disarray and replacement-type fibrosis.

### ACM (right ventricular)

ACM is a rare genetically determined disorder of the myocardium with a prevalence of 0.01%–0.05% in the general population [30–33]. In the consecutive series of SD of the Veneto Region in Italy, ACM is the second most frequent cause of SD in the young and the first among athletes [34]. This cardiomyopathy shows a peculiar age and gender-related penetrance of the phenotype, with SD typically occurring during adolescence or early adulthood, mainly in males, even as first manifestation of the disease. The most common inheritance pattern is autosomal dominant with pathogenic mutations in desmosomal genes encoding for plakoglobin (*JUP*), desmoplakin (*DSP*), plakophilin (*PKP-2*), desmoglein-*DSG2* and desmocollin-*DSG2* [31,32,35–38]. Although the majority of mutations for ACM occurs in genes coding for desmosomal proteins, some mutations are localized in genes only partially linked to junctional complexes. Only isolated reports showed causal mutations in non-desmosomal genes, such as transmembrane protein 43 (*TMEM43*), desmin (*DE*S), titin (*TTN*), Lamin A/C (*LMNA*), phospholamban (*PLN*), αT-catenin (*CTNNA3*) and *CDH2*, encoding cadherin 2. Genetic screening can provide a positive result in up to 50% of familial cases. Genotype-phenotype correlations are still limited in ACM. More than one mutation with different prognostic value may be present in the same individual, with important consequences in terms of genetic counselling [39].

**Figure 5.**
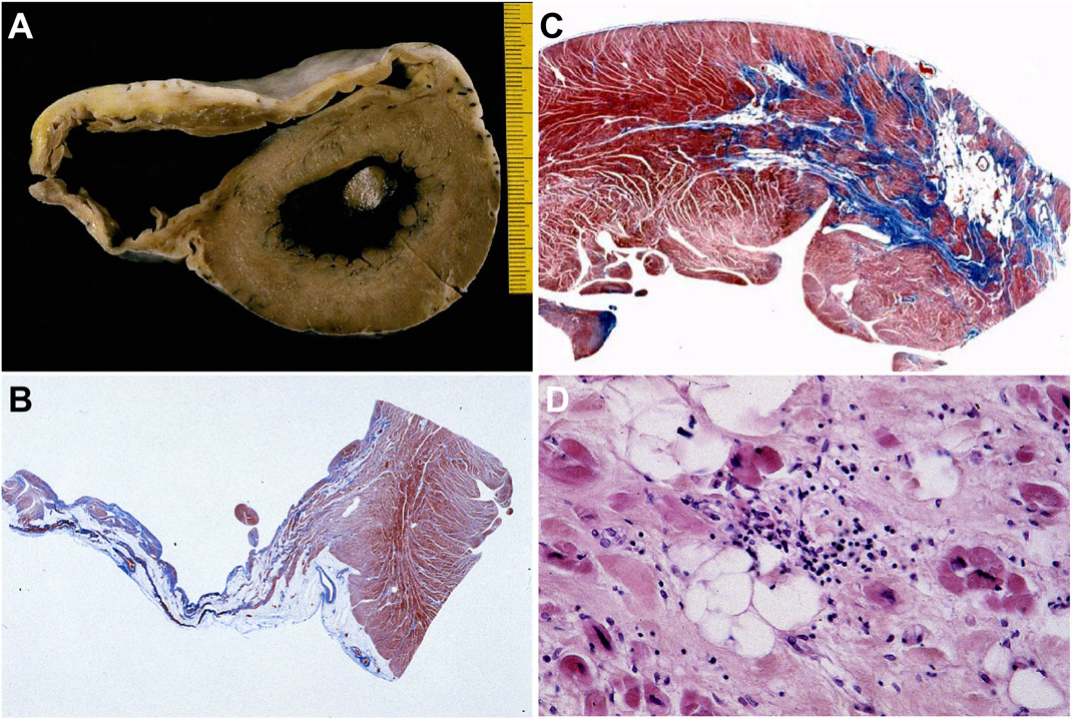
Arrhythmic sudden death due to arrhythmogenic cardiomyopathy (diffuse form) in a 14-year-old boy, during a soccer play. (A) Cross-section of the heart showing the presence of anterior and posterior aneurysms as well as patchy involvement of the left ventricular free wall, postero-lateral region; (B) Histology of the aneurysmal postero-inferior wall: note the loss of myocardium with fibro-fatty replacement (Trichrome Heidenhain, ×2.5); (C) Histology of the left ventricular free wall in the areas of fibro-fatty replacement (Trichrome Heidenhain, ×5); (D) Adipogenesis in areas of myocyte injury (HE, ×200).

The main histopathological feature of ACM is the progressive loss of ventricular myocardium and the fibro-fatty replacement from epi- to endocardium. The so-called triangle of dysplasia (inflow tract or subtricuspid region, apex, outflow tract or infundibulum) is the region more frequently involved by the pathological process. Right ventricular aneurysms in this area are considered patognomonic of the disease [34,40]. Macroscopically, the fibrous or fibro-fatty scars appear as yellowish or whitish areas sometimes reaching transmural extension, resulting in a dilated ventricle with a thinned, parchment-like and translucent wall (Figures 4 and 5). Recently, many studies extended the morphological spectrum of the disease, describing biventricular or left ventricular dominant forms [41–43], in which fibro-fatty or fibrous scars are typically located in the epicardial layers of the postero-lateral free wall. The interventricular septum is usually spared from the disease. The term ACM, instead of arrhythmogenic right ventricular cardiomyopathy, has been then proposed to include these different clinical-pathological subtypes of the disease (classical right ventricular, left dominant or biventricular phenotype). The involvement of the left ventricle by the fibrous substitution can be detected during life only by imaging tools, such as contrast-enhanced cardiac magnetic resonance (CMR). Different studies suggested then the crucial role of CMR as to include it in an updated version of the diagnostic criteria for ACM [44,45]. The gross findings for ACM may be difficult to recognize or even absent in some cases (“concealed” or segmental forms) and only histopathological examination will reveal features of typical ACM. Thus, both ventricles should be extensively sampled for histology in all cases of SD [1,2].

At histology, myocardial substitution by fibrous or fibro-fatty tissue, starting from the subepicardium is the pathognomonic feature (Figures 4 and 5). Fatty infiltration alone is not a distinctive element of the disease, the presence of replacement-type fibrosis and the myocyte degenerative changes are essential to obtain a clear-cut diagnosis. [46]. The finding of focal myocardial inflammation (mostly T-lymphocytes and macrophages) is reported in nearly 75% of the cases and could contribute to the worsening of the electrical instability and the genesis of life-threatening arrhythmias [40].

### Myocarditis

Myocarditis is defined as an inflammatory disease of the myocardium established by histology, immunology and immunohistochemistry. Myocarditis can present in many different ways, including SD, particularly in the young population [6–9].

**Figure 6.**
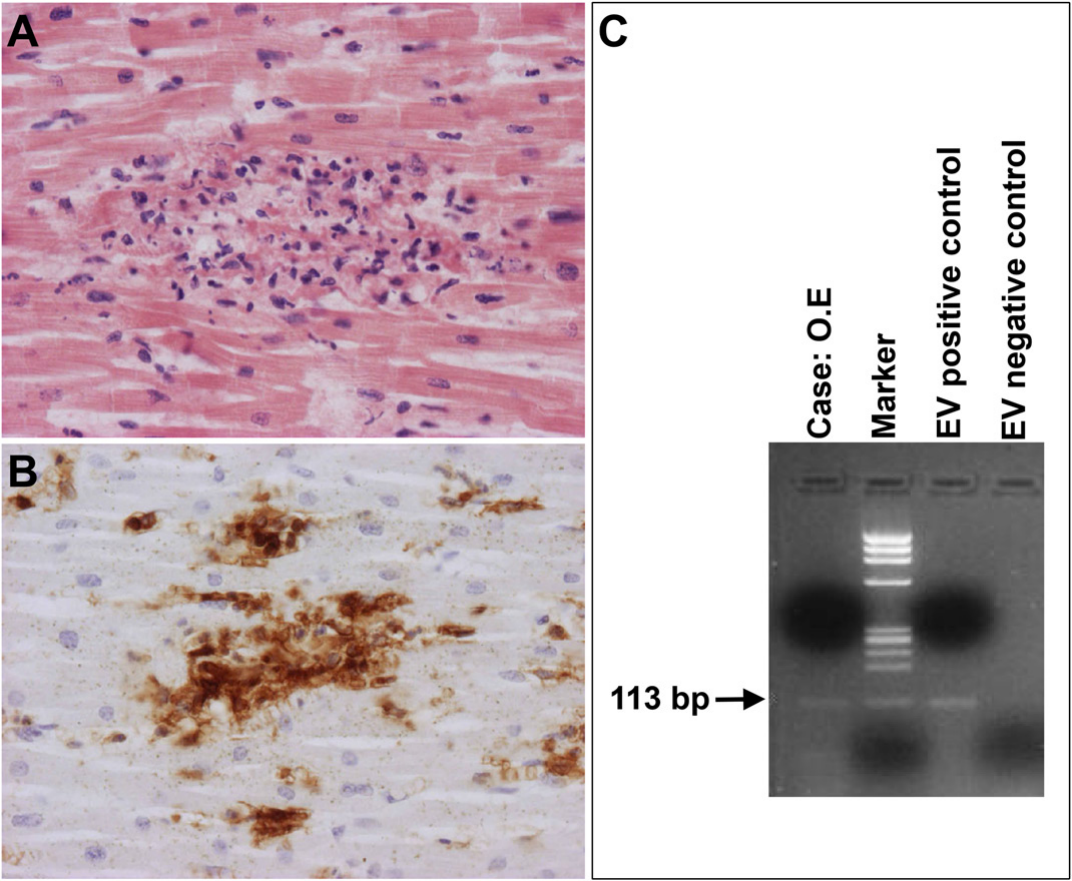
Arrhythmic SD due to lymphocytic myocarditis. (A) Focus of inflammatory infiltrates associated with myocyte necrosis (HE stain, ×200); (B) At immunohistochemistry, the inflammatory infiltrate is rich in T-lymphocytes (CD3 immunostaining, ×200); (C) PCR-proved lymphocytic myocarditis due to enterovirus infection.

Grossly, the heart may appear normal, but at histological analysis, interstitial edema, focal or diffuse inflammatory infiltrates, predominantly lymphocytic with associated myocyte necrosis and replacement-type fibrosis can be observed in the ventricular myocardium (Figure 6). Even mild myocardial inflammation can trigger ectopic automatism and be sufficient to dysregulate the electrical function of the heart. Release of cytokines, with interstitial edema and patchy necrosis, may jeopardize the depolarization-repolarization phases of the myocardium, thus favoring trigger activity. However, in the absence of myocyte necrosised, small foci of inflammatory cells (even after immunohistochemistry), are not sufficient to reach the diagnosis of myocarditis. In the subacute-chronic stages of myocarditis, varying degrees of replacement-type fibrosis might also account for electrical instability, with or without persistent inflammation.

Among the various causes of myocarditis (infections, allergens, drugs, toxic agents, autoimmune or hypersensitivity reaction), viral infection is the most frequent etiology in SD cases [9,47,48]. In lymphocytic myocarditis, molecular techniques such as polymerase chain reaction (PCR) on both myocardial tissue (even paraffin-embedded) and blood are the gold standard for the diagnosis of viral myocarditis. A large spectrum of viruses can be found at molecular analysis; moreover the viral genome load should be quantified in order to exclude the presence of innocent bystanders (such as Parvovirus B19) [9,47,48].

Clinical manifestation of myocarditis is highly variable, ranging from mild symptoms of chest pain and palpitations associated with transient ECG changes to life-threatening cardiogenic shock and ventricular arrhythmias. Endomyocardial biopsy is recommended in clinical suspected myocarditis, after exclusion of coronary artery disease [48–50]. Molecular investigation should be included to look for (or rule out) a causative viral agent. Cardiac sarcoidosis can mimic ACM or myocarditis and be a cause of ventricular arrhythmias and SD, even as the first manifestation. Toxicologic investigation in suspected myocarditis is mandatory to exclude possible unnatural triggers, particularly in young adults and competitive athletes [1,2].

### Non-structural cardiomyopathies (channelopathies)

In our experience, about 20% of the SDs in the young remain unexplained after complete autopsy including gross and histological examination of the heart and toxicological analysis (“mors sine materia”) [6–8]. Part of these unexplained SDs are considered to be caused by cardiac ion channel diseases (channelopathies), such as LQT syndrome, Brugada syndrome, CPVT and SQT syndrome. At difference from the structural cardiac diseases, the heart is morphologically normal, and the defect is at molecular level, due to pathogenic mutations in genes encoding cardiac ion channel proteins. The ion channel diseases share an unstable electrical cardiac activity at risk of ventricular fibrillation [51,52]. Overt or latent functional gene variants causing these disorders, in particular LQT syndrome, have also been associated with the sudden infant death syndrome [53].

In case of SD with normal heart, is crucial the correlation with clinical data, in particular ECG performed during life, a personal and family history and information of the circumstances of death (at rest, during effort or emotion, various triggers, etc.). As a consequence, first-degree relatives should undergo clinical screening and possible genetic testing.

Performing a detailed postmortem investigation, with the employment of molecular biology techniques (so called “molecular autopsy”), represent the only opportunity to reach the correct diagnosis in the victim and relatives [54] as recommended in the guidelines for autopsy investigation of SD proposed by the Association for European Cardiovascular Pathology [2]. Adequate sampling for further laboratory test in SD cases is recommended. Stored materials should be sent to specialized local or regional centers to perform the necessary toxicology, chemistry, microbiology and genetic testing [2].

In some cases, SD can still remain unexplained, even after a thorough investigation of genes known to be related to the abovementioned syndromes. Idiopathic ventricular fibrillation may represent another cause of SD with normal heart to be taken into account, with arrhythmias triggered by a dysfunction of the distal Purkinje fibers [55–59].

Many times, a genetic explanation will be found in the “mors sine materia” cases, however, caution is needed due to the difficult interpretation of the pathogenic significance of gene mutations.

## Conclusion

As SD may be the first manifestation of the underlying cardiomyopathy, the autopsy should include thorough cardiac examination according to national and international protocols.

The updated European guidelines for autopsy investigation of SD [2] recommend to conclude the final postmortem report with a clear clinical-pathological synthesis (epicrisis), including a certain/probable/possible cause, useful in case of inherited cardiovascular disease, to trigger a clinical and genetic investigation of first-degree family members. A multidisciplinary approach by a cardiologist, geneticist, pathologist, sport physician and general practitioner is mandatory for the proper care of family members.
